# Effects of Acute Cortisol Administration on Perceptual Priming of Trauma-Related Material

**DOI:** 10.1371/journal.pone.0104864

**Published:** 2014-09-05

**Authors:** Elena Holz, Johanna Lass-Hennemann, Markus Streb, Monique Pfaltz, Tanja Michael

**Affiliations:** 1 Division of Clinical Psychology and Psychotherapy, Department of Psychology, Saarland University, Saarbrucken, Germany; 2 Division of Psychiatry and Psychotherapy, University Hospital Zurich, Zurich, Switzerland; Sapienza University of Rome, Italy

## Abstract

Intrusive memories are a hallmark symptom of posttraumatic stress disorder (PTSD). They reflect excessive and uncontrolled retrieval of the traumatic memory. Acute elevations of cortisol are known to impair the retrieval of already stored memory information. Thus, continuous cortisol administration might help in reducing intrusive memories in PTSD. Strong perceptual priming for neutral stimuli associated with a “traumatic” context has been shown to be one important learning mechanism that leads to intrusive memories. However, the memory modulating effects of cortisol have only been shown for explicit declarative memory processes. Thus, in our double blind, placebo controlled study we aimed to investigate whether cortisol influences perceptual priming of neutral stimuli that appeared in a “traumatic” context. Two groups of healthy volunteers (N = 160) watched either neutral or “traumatic” picture stories on a computer screen. Neutral objects were presented in between the pictures. Memory for these neutral objects was tested after 24 hours with a perceptual priming task and an explicit memory task. Prior to memory testing half of the participants in each group received 25 mg of cortisol, the other half received placebo. In the placebo group participants in the “traumatic” stories condition showed more perceptual priming for the neutral objects than participants in the neutral stories condition, indicating a strong perceptual priming effect for neutral stimuli presented in a “traumatic” context. In the cortisol group this effect was not present: Participants in the neutral stories and participants in the “traumatic” stories condition in the cortisol group showed comparable priming effects for the neutral objects. Our findings show that cortisol inhibits perceptual priming for neutral stimuli that appeared in a “traumatic” context. These findings indicate that cortisol influences PTSD-relevant memory processes and thus further support the idea that administration of cortisol might be an effective treatment strategy in reducing intrusive reexperiencing.

## Introduction

Intrusive memories are a hallmark symptom of posttraumatic stress disorder (PTSD). They usually consist of brief, sensory fragments of the event and are easily triggered by a variety of internal and external stimuli [1], [2]. In contrast to ordinary autobiographical memories, intrusions are involuntarily and unintentionally retrieved, lack context information and are accompanied by a sense of “nowness”, i.e. the feeling that the event is something happening in the “here and now” rather than stemming from the past [3], [4]. Intrusive memories reflect excessive and uncontrolled retrieval of the traumatic memory, which usually retains its vividness and power to evoke distress for decades or even a life time. Thus, the reduction of intrusive memories is one of the main aims of PTSD therapy and much research has focused on the development and therapy of intrusive memories.

Strong perceptual priming has been proposed to be one mechanism that leads to the development of intrusive memories [5]. Ehlers and Clark [5] suggested that trauma survivors who acquire strong priming for stimuli that they encountered during the traumatic event, have a reduced perceptual threshold for these stimuli and thus they are more likely to detect potential triggers of intrusive memories in their environment. Indeed, clinical observations suggest that triggers of intrusions often match salient sensory characteristics of stimuli that were present at the scene of the trauma. These stimuli do not necessarily bear a meaningful relationship with the trauma and may only be temporally associated with the traumatic event.

Two lines of research support the role of perceptual priming in the development of intrusive memories. First, clinical studies have shown that PTSD patients show greater priming for trauma-related material compared to control participants in word-stem completion or perceptual identification tasks [6], [7], [8]. Importantly, priming also predicted PTSD severity at 6 month follow-up [6], [7]. Kleim and colleagues [9] further found that trauma survivors with PTSD show perceptual processing advantages in identifying blurred trauma-related pictures compared to trauma survivors without PTSD.

The second line of research employed analogue studies to investigate the role of perceptual priming in the development of intrusive memories [10], [11], [12], [13]. Originally, Ehlers and colleagues [10] developed an analogue paradigm to study perceptual priming for visual stimuli in a “traumatic” context. Employing picture stories, the paradigm investigates priming for neutral objects which occur just before something “traumatic” happens. Modified versions of this paradigm have been applied in several studies, each showing enhanced priming for neutral stimuli in a “traumatic” context [11], [12], [14]. In addition, greater priming also predicted intrusive memories of the picture stories [10], [11], [12]. Thus, there is converging empirical evidence suggesting that perceptual priming plays a role in the development and maintenance of intrusive memories.

Recently, the steroid hormone cortisol has been proposed as a pharmacological option to reduce intrusive memories [15], [16]. Cortisol influences a variety of peripheral and central physiological processes. Importantly, high cortisol levels have been shown to inhibit memory retrieval in healthy subjects and animals (for a review see [17]). Exogenous cortisol administration, cortisol reactions in response to stress (e.g. [18]) and high basal cortisol levels (e.g. [19]) all lead to impaired memory retrieval (but see [20], [21], [22] for contrary findings). Even though cortisol secretion is related to stress, it has to be noted that PTSD is associated with low basal cortisol levels [23], [24], [25], [26], [27] (but see [28], [29] for contrary findings).

Cortisol may serve as a pharmacological support for reducing intrusive memories in PTSD patients by inhibiting the excessive retrieval of traumatic memories [16]. To our knowledge, there is only one study investigating the effects of cortisol administration on intrusive reexperiencing: In a pilot study with 3 PTSD patients Aerni et al. [30] showed that exogenous cortisol administration over 1 month had a beneficial influence on reexperiencing symptoms. However, the results were not very conclusive: Cortisol reduced intrusion intensity in two patients, but had no effect on intrusion frequency, while it reduced nightmare frequency in the third patient, but had no effect on intrusion intensity or frequency.

Further support for the cortisol hypothesis comes from studies employing single high doses of hydrocortisone to traumatized patients in order to prevent the development of PTSD: Administering hydrocortisone to intensive care patients lead to a decrease in the incidence of subsequent PTSD [31], [32], [33]. Delahanty and colleagues [34] showed that repeated cortisol administration (over 10 days) prevented PTSD in traumatic injury patients.

One problem in the application of cortisol in PTSD therapy is that the memory modulating effects of cortisol have primarily been shown for explicit declarative memory processes (for a review see [35]). There are only a few studies investigating the effects of cortisol on implicit memory processes and these showed mixed results [36], [37], [38], [39]. However, as described above, intrusions are thought to rely on implicit memory processes, e.g. perceptual priming. To our knowledge there is only one study investigating the effects of cortisol on perceptual priming for neutral stimuli and in this study cortisol did not affect perceptual priming for neutral material [36]. Thus, it is not clear whether cortisol influences perceptual priming for neutral stimuli in a “traumatic” context.

In this study, we aimed to investigate whether cortisol inhibits perceptual priming for neutral stimuli that appear in a “traumatic” context. The paradigm we used is a modified version of the paradigm by Sundermann and colleagues [12].

In our paradigm we addressed two limitations of the previously developed paradigms: We decided to use a between subject design, because the previously employed within subject designs might lead to carry over effects from “traumatic” to neutral stories. Finally, to date, the priming effect has only been tested directly after presentation of the “traumatic”/neutral stories. However, if strong perceptual priming for neutral stimuli in a “traumatic” context functions as a way of developing intrusive memories, this effect should persist over time. Thus, we decided to test priming after a longer time period (24 hours).

In our study two groups of healthy volunteers (N = 80 per group) watched either neutral or “traumatic” picture stories on a computer screen. Neutral objects were presented in between the pictures in each group. Memory for these neutral objects was tested on the next day with a perceptual priming task and an explicit memory task. Prior to recall half of the participants in each group received 25 mg of cortisol, the other half received placebo. For the placebo group we expected participants in the “traumatic” condition to show more perceptual priming for the neutral objects than participants in the placebo/neutral stories condition. Due to the retrieval inhibiting effect of cortisol administration, this effect should be reduced in the cortisol group. More precisely, we expected cortisol to reduce the enhanced perceptual priming effect in the “traumatic” picture stories condition.

## Methods

### Participants

Participants were 160 healthy students (80 women, 80 men) with a mean age of 22.83 (SD 3.02, range 18–34) at Saarland University, Germany, who responded to notices offering 30 Euros for participation in a psychological experiment. Participation was restricted to healthy, non-smoking students with a body mass index of 20–25 kg/m^2^. To minimize the influence of menstrual cycle phase on hormonal status only women with a regular use of monophasic oral contraceptives were included. Exclusion criteria were a recent history of systemic or oral cortisol therapy, any pharmacological treatment, any current DSM-IV axis I disorder, severe acute or chronic physical disease, pregnancy and lactation, and participation in a pharmacological study within the past 3 months. We also required participants to refrain from physical exercise, alcohol, and caffeinated drinks 3 h prior to each of the two experimental sessions. The experimental session took place between 2 p.m. and 6 p.m. to control for the diurnal cycle of cortisol. All participants gave written informed consent. The research was approved by the ethical committee of the medical association of Saarland.

### Materials and Methods

The experimental software for all parts of the experiment was programmed with E-Prime (E-Prime 2.0). Picture stories and memory tests were presented on a 19″ computer screen of a Dell computer (Dell Precision T1500).

#### Picture stories

We used a modified version of the design and materials from Sundermann [12]. The main difference between the two paradigms is that we employed a between-subject design. In our study, participants watched either “traumatic” picture stories or neutral picture stories in order to minimize carry over effects. Furthermore, perceptual priming and explicit memory for the neutral objects was tested 24 h after participants watched the picture stories in order to test whether the perceptual priming effect persists over time.

Participants were randomly assigned to watch either five “traumatic” picture stories or five neutral picture stories in randomized order. Participants watched the “traumatic” or the neutral picture stories on a computer screen, and listened to a running commentary of what was happening in the story. Each story started with three pictures introducing the main characters (see Table 1). In the “traumatic” stories, the plot unfolded in a “traumatic” way over four further pictures (e.g. a car accident in which the main character dies), whereas the plot continued neutrally over four pictures in the neutral story (e.g. a walk in the park). For every “traumatic” picture story a matched neutral picture story was generated. Thus, the first three pictures (and the commentary) in every “traumatic” picture story and its corresponding neutral picture story were the same.

**Table 1:** Example of a neutral and a “traumatic” picture story including the presented priming objects.

Picture story example	Neutral story	„Traumatic“ story
**Picture 1**	This is Susanne Kaufmann. She's 36 years old and works in a big investment company.	This is Susanne Kaufmann. She's 36 years old and works in a big investment company.
**Picture 2**	After a long working day with several meetings and numerous phone calls, she is finally getting ready to go home.	After a long working day with several meetings and numerous phone calls, she is finally getting ready to go home.
**Picture 3**	She gets in her car still with a lot of things on her mind. She also starts thinking about her plans for the evening.	She gets in her car still with a lot of things on her mind. She also starts thinking about her plans for the evening.
**Priming objects**	adhesive tape dispenser, handbag	adhesive tape dispenser, handbag
**Picture 4**	Susanne is going to spend the evening with her fiancé Peter. They meet up at the park and go for a walk along the lake.	Suddenly a cyclist appears just in front of her car. Susanne hits the breaks hard causing the car to swerve on the wet road and crash into a concrete pier.
**Priming objects**	personal organizer, cellphone	personal organizer, cellphone
**Picture 5**	As it is a fairly bright evening, the two of them decide to hire a rowing boat and take a trip around the lake.	The paramedics arrive quickly. Susanne is in a terrible state as the collision has caused severe damage to her head, face, and vital organs.
**Priming objects**	stapler, puncher	stapler, puncher
**Picture 6**	Later that evening Susanne and Peter go out for dinner at a restaurant that they often go to.	They rush her to the hospital trying to resuscitate her on the way there and to save her life.
**Picture 7**	Hungry from the boat trip they enjoy their meal and a glass of wine while they chat about their day.	But the injuries are too serious. Susanne dies from a brain bleeding.

Neutral objects (the same objects in the “traumatic” and its corresponding neutral picture stories) were presented in pairs prior to the onset of pictures 4, 5, and 6. We decided to present the neutral objects at these points, because clinical observations suggest that intrusive memories usually consist of sensory memories of stimuli that were present before the traumatic event happened or immediately before the moment of the largest emotional impact. Thus, our neutral objects were presented before the onset of the trauma (picture 4) and the highest emotional impact (picture 5, assault of main character; picture 6, death of main character). Neutral objects were selected to fit into the context of the “traumatic” and its corresponding neutral story (e.g. office setting – stapler, puncher).

The timing of presentation of each picture story was as follows: each picture was shown for 14 seconds, the corresponding narration started with a 1 second delay to each picture. Each picture was followed by an inter-trial interval (blank screen) for 1 second. Neutral objects (primes) were presented side by side at the center of the screen for 750 ms after the preceding inter-trial intervals.

In order to make the material as realistic as possible, the stories were made up from documentaries and feature films starring actors and actresses who were not well known. The cultural background of the stories was embedded in a “western” context so that the participants (who all lived in a similar context) could easily identify with the main characters. The “traumatic” and neutral picture stories were matched for the number of males and females occurring in them, and whether the event happened indoors or outdoors.

Participants were told that the purpose of the experiment was to test how picture stories influenced people’s emotions. Furthermore, the experimenter explained to them that the stories are based on events that actually happened and asked them to pay attention to the story and imagine that they were an eyewitness of the scenes.

### Memory measures

Memory for objects shown in the picture stories was tested with a blurred object identification task (assessing perceptual priming) and a recognition task (assessing explicit memory).

Perceptual priming task: Participants were told that this task was unrelated to the picture stories in the first experimental session. They were informed that the task was about the impact of cortisol administration on the ability to identify blurred objects. To prevent that participants noticed that the task was a memory test, only 15 (of 30) primed objects were included. The majority of objects (N = 20) were unprimed (distractors) and had not been presented in the picture stories.

Primed objects were blurred with a Gaussian filter, allowing approximately 50% identification in pilot participants with no prior exposure to the picture stories. Unprimed (distractor objects) objects were blurred slightly less so that their baseline identification rate was about 60%. This was done to ensure that each participant would identify at least a few distractor objects, reducing the chance that participants would notice that the task tests memory performance for stimuli from the picture stories.

All blurred pictures were presented individually on a computer screen for a maximum of 2 seconds. They were presented in a random order that varied with each participant. Participants were instructed to look at the pictures and to guess what the objects might be, working as quickly and as accurately as possible. They had to type in their guess on a keyboard. To prevent further processing of the pictures and to encourage participants to actually type in their first guess, objects disappeared from the screen as soon as participants typed the first letter. After typing their answer, participants moved on to the next object by pressing the ENTER key.

Recognition task: This task was included to estimate possible influences of explicit memory on the results of the perceptual priming task. For each “old” object from the picture stories a corresponding new object (corresponding object) was chosen. These corresponding objects matched the objects from the picture stories in size and object type (e.g., if the old object [from the picture stories] was a watch, a different watch of approximately the same size served as a corresponding object). Objects were presented on a computer screen in a successive, random order. Participants were asked to indicate whether or not they had previously seen the object in the stories by pressing the keys “a” for “alt” (old in German) and “n” for “neu” (new in German) on the computer keyboard.

### Priming objects, distractor objects and corresponding objects

**Figure 1:**
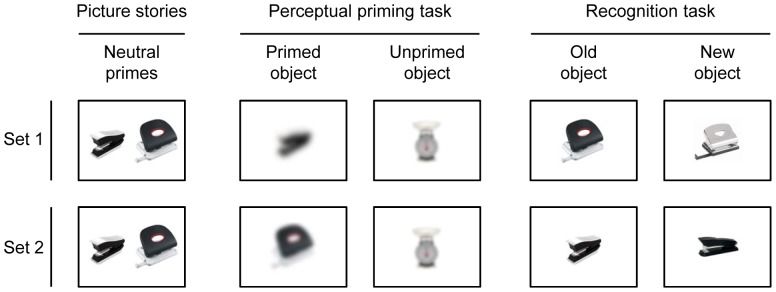
Examples of the objects presented in the picture stories, perceptual priming task, and recognition task.

All objects in both tasks had a resolution of 600*600 pixels. The 30 priming objects of the picture stories were randomly assigned to two sets of 15 pictures each. Half of the participants saw the primes of set 1 in the blurred picture identification task and the priming objects of set 2 in the recognition task and vice versa. This was done to ensure that participants saw each priming object only once either in the blurred picture identification task or in the recognition task. Figure 1 shows examples of the objects presented in the picture stories and in both memory tasks.

### Measurement of cortisol

Cortisol data was collected using Salivette tubes (Sarstedt). The participant first placed a cotton swab provided in each Salivette tube in his or her mouth and gently chewed on it for about one minute. The swab was then placed back in the tube. Tubes were kept at −20°C until analysis. Saliva cortisol was analysed at the cortisol laboratory of the University of Trier, Germany. After thawing the saliva samples for biochemical analysis, the fraction of free cortisol in saliva was determined using a time-resolved immunoassay with fluorometric detection, as described in detail elsewhere [40]. Salivary cortisol of one participant in the “traumatic” stories/cortisol condition and one participant in the “traumatic” stories/placebo condition was missing.

### Procedure

The study took place at the laboratories of the Department of Clinical Psychology and Psychotherapy of Saarland University, Germany. Participation included three appointments: an initial screening session to determine eligibility and two experimental sessions. Both experimental sessions were run between 02.00 p.m. and 06.00 p.m.

Participants were randomized with respect to sex by an independent person to one of the four conditions (“traumatic” stories/cortisol, “traumatic” stories/placebo, neutral stories/cortisol, neutral stories/placebo).

### Screening interview

If participants fulfilled the inclusion criteria in a short telephone screening, they were invited to a first laboratory appointment. After reading a study information sheet, participants gave written informed consent. Thereafter, inclusion and exclusion criteria where checked again and participants completed a demographic questionnaire.

### Experimental session 1

After participants arrived for the first experimental session, they were seated in a sound attenuated cabin and watched the neutral or “traumatic” picture stories. Audio commentaries were presented via headphones. After each picture story, participants rated the valence and arousal of the picture story on a visual analogue scale (0–100). Thereafter, participants determined the start of the subsequent picture story by pressing the SPACE button. After the last valence and arousal rating, an appointment for the second experimental session (24 h after the first session) was scheduled and participants left the laboratory. Experimental session 1 lasted approximately 20–30 minutes.

### Experimental session II

All participants, blinded to treatment status, received either 25 mg oral cortisol (Hydrocortison, Jenapharm) or placebo (P-Tabletten, Liechtenstein) one hour before memory measures were taken. Saliva samples were collected immediately before and 1 h after cortisol or placebo administration, when the cortisol concentration is supposed to reach its peak (compare [41]). One h after cortisol or placebo administration, the two memory tests were carried out. Participants first completed the blurred picture identification task, followed by the recognition task. The two memory tasks took about 10–15 minutes to complete. Finally, participants were debriefed and received 30 Euros for their participation. They could furthermore ask and discuss questions on the study design and goals of the study.

### Data Analysis and Reduction

#### Perceptual priming task

Two independent raters (Cohens Kappa = 1.00) judged the priming objects as being correctly or incorrectly identified. Priming objects were scored as correctly identified if participants typed in the correct object name or a close synonym (defined a priori to the experiment). For each participant, percentage of correctly identified priming objects was calculated. Effects of cortisol administration on perceptual priming for “traumatic” and neutral picture stories was analyzed with a 2*2 between subject ANOVA with drug intake (cortisol vs. placebo) and story content (“traumatic” vs. neutral) as independent variables. Set condition, posttreatment cortisol levels and sex of the participants were controlled for by adding them as covariates. Adding the covariates did not change the results concerning the effects of drug intake and story content on perceptual priming.

#### Distractor objects

Identification performance for the distractor objects was calculated in the same way as identification performance for the priming objects. To rule out purely perceptual factors of cortisol on identification performance for blurred objects we subjected the distractor objects to a 2*2 between subject ANOVA with drug intake (cortisol vs. placebo) and story content (“traumatic” vs. neutral) as independent variables. Set condition, posttreatment cortisol levels and sex of the participants were controlled for by adding them as covariates. Adding the covariates did not change the results concerning the effects of drug intake and story content on identification of distractor objects.

#### Recognition memory

Data analysis of the object recognition task followed signal detection theory (SDT). This analysis allows computing of sensitivity (d’) and response bias (c) scores. Sensitivity measures how well participants discriminated between old and new objects. Response bias assesses participants’ implicit readiness to identify or reject objects as old objects. Effects of cortisol administration on sensitivity and response bias for “traumatic” and neutral picture stories were analyzed with a 2*2 between-subject ANOVA with drug intake (cortisol vs. placebo) and story content (“traumatic” vs. neutral) as independent variables. Set condition, posttreatment cortisol and sex of participants were controlled for by adding them as covariates into the model. Adding the covariates did not change the results concerning the effects of drug intake and story content on recognition memory.

## Results

### Manipulation Checks

#### Validity of picture stories

Participants in the “traumatic” stories groups rated the stories as more aversive (M = 24.11 (SD 13.78); *t*(158) = 20.48, *p*<.001) and more arousing (M = 57.23 (SD 16.03); *t*(158) = −8.08, *p*<.001) than participants in the neutral stories groups (aversive: M = 67.77 (SD 13.17), arousing: M = 33.81 (SD 20.39)).

#### Cortisol administration

The manipulation check for cortisol revealed a significant interaction of drug intake (cortisol vs. placebo) and time (pre-treatment vs. post-treatment) (*F* (1, 154) = 655.21, *p*<.001, *η* ^2^ = .81). Before treatment there was no difference in cortisol concentrations between groups (*p* = .55). One h after treatment, cortisol concentrations were significantly increased in the two cortisol groups, compared with the two placebo groups (*p*<.001). There was also a significant three-way-interaction between drug intake, time, and story context. Participants in the “traumatic” stories/cortisol condition had higher post treatment cortisol scores than participants in the neutral stories/cortisol condition (F (1,154) = 8.97, p = .003, *η* ^2^  = .05). However, cortisol levels in both groups were very high. Data are presented in Table 2.

**Table 2:** Salivary Cortisol in nmol/l at pre- and posttreatment in the four conditions.

	Neutral stories		„Traumatic“ stories	
	Placebo	Cortisol	Placebo	Cortisol
	M (SD)	M (SD)	M (SD)	M (SD)
Salivary Cortisol pre-treatment	3.87 (2.17)	3.62 (2.75)	4.22 (2.44)	3.99 (2.48)
Salivary Cortisol post-treatment	3.21 (1.70)	73.29 (34.23)	3.09 (1.32)	91.83 (17.79)

#### Cortisol effects on perceptual priming

**Figure 2:**
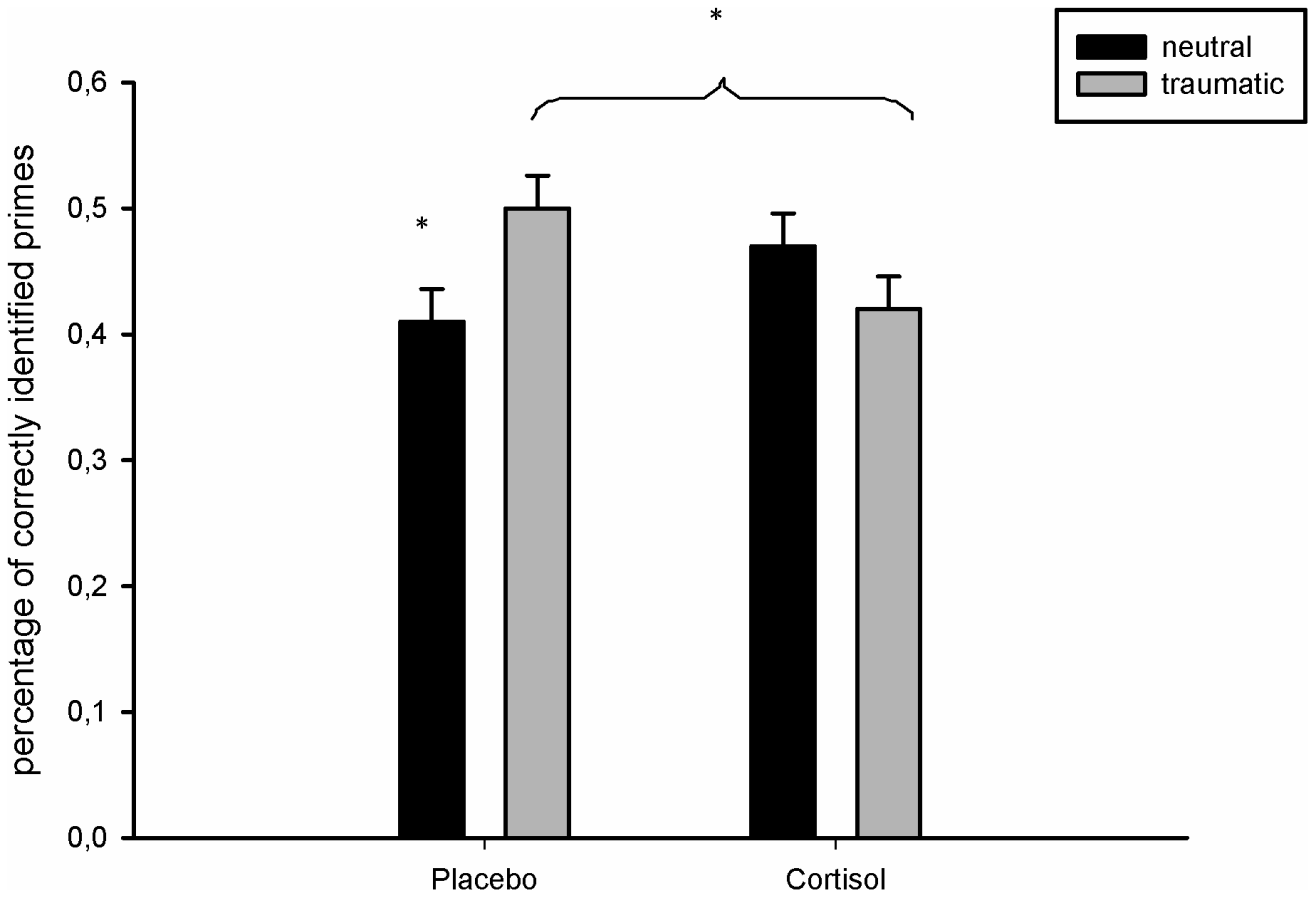
Cortisol effects on perceptual priming: In the placebo group participants in the “traumatic” stories condition showed more perceptual priming for the neutral objects than participants in the neutral stories condition, indicating a strong perceptual priming effect for neutral stimuli presented in a “traumatic” context. In the cortisol group this effect was not present: Participants in the neutral stories and participants in the “traumatic” stories condition in the cortisol group showed comparable priming effects for the neutral objects.

There was no significant main effect of story content or drug intake on perceptual priming. However, as expected there was a significant interaction between drug intake (cortisol vs. placebo) and story content (“traumatic” vs. neutral) (*F* (1,153) = 5.64, *p* = .02). Participants in the “traumatic” stories/placebo condition showed significantly more perceptual priming than participants in the neutral stories/placebo condition (*p* = .02). Furthermore, participants in the “traumatic” stories/cortisol condition and participants in the “traumatic” stories/placebo condition differed significantly in the percentage of correctly recalled primes (*p* = .048). Participants in the “traumatic” stories/cortisol condition and participants in the neutral stories/cortisol condition did not differ significantly from each other (p = .24). Participants in the neutral stories/placebo condition and neutral stories cortisol condition also did not differ significantly from each other (p = .11). Figure 2 illustrates these findings.

#### Cortisol effects on distractor objects

There was no significant main effect of story context (F (1,153) = .001, p = .97) and drug intake (F (1, 153) = .01, p = .91) on identification performance for distractor objects. The interaction between drug intake and story context was also not significant (F (1,153) = 1.47, p = .23).

#### Cortisol effects on recognition memory

There was no significant main effect of story context (d’: *F* (1, 153) = 1.00, *p* = .32, c: *F* (1,153) = 1.00, *p* = .32) or drug intake (d’: *F* (1,153) = .002, *p* = .96, c: *F* (1, 153) = .06, *p* = .81) on sensitivity or response bias scores. The interaction between story context and drug intake was also not significant for sensitivity (*F* (1,153) = .29, *p* = .59) and response bias (*F* (1,153) = .01, *p* = .95). Data are presented in Table 3.

**Table 3:** Sensitivity (d’) and response bias (c) in the recognition memory test in the four conditions.

	Neutral stories		„Traumatic“ stories	
	Placebo	Cortisol	Placebo	Cortisol
	M (SD)	M (SD)	M (SD)	M (SD)
Sensitivity (d‘)	0.10 (0.99)	0.07 (1.16)	−0.18 (0.99)	0.01 (1.19)
Response bias (c)	−0.16 (0.67)	0.01 (0.91)	−0.02 (0.85)	0.17 (0.91)

## Discussion

In the present study we could replicate and extend findings on enhanced perceptual priming in a “traumatic” context [10], [11], [12], [14]: Participants in the “traumatic” stories/placebo condition showed more perceptual priming than participants in the neutral stories/placebo condition. Importantly, we found that this perceptual priming effect persists, when memory is tested one day after learning. As mentioned above, enhanced perceptual priming in a “traumatic” context only provides a suitable explanation for intrusive memories if it persists over time. The findings of the placebo group thus provide further support for the role of perceptual priming in intrusive memories.

Furthermore, the results of the present study show for the first time that exogenous cortisol administration does reduce perceptual priming for neutral objects in a “traumatic” context. As expected, enhanced priming in a “traumatic” context was not present in the cortisol conditions: Participants who watched “traumatic” stories and received cortisol did not show enhanced perceptual priming as compared to participants in the neutral stories/cortisol condition. Thus, cortisol seems to inhibit the retrieval of perceptually primed material in a “traumatic” context. Cortisol has been proposed as a potential pharmacological help in reducing intrusive memories. Our data show that cortisol administration does indeed inhibit the retrieval of previously perceptually primed material in a “traumatic” context. Up to date the effects of cortisol have been primarily shown for explicit hippocampus dependent memory processes. Studies investigating implicit memory processes yielded mixed results [35]. The inconsistent influence of cortisol on implicit memory processes has been attributed to the degree of the involvement of the hippocampus in these memory processes. Cortisol receptors are mainly distributed in the hippocampus and medial prefrontal cortex and whereas explicit memory processes are thought to rely on these structures (e.g. [42]), implicit memory processes are often thought to be independent of the hippocampus. However, there are also some studies showing a hippocampal involvement in priming studies (e.g. [43]). Also, in our study we found an influence of cortisol only in the “traumatic” stories condition. There are several studies showing that cortisol mainly affects emotional memory processes [17]. This has been attributed to an interplay of the amygdala and the hippocampus. It is likely that our “traumatic” stories also led to a strong amygdala activation. Thus, we believe that the mechanisms underlying the effects of cortisol on perceptual priming in a “traumatic” context might be similar to those underlying the effects of cortisol on explicit memory processes.

Interestingly, cortisol did not have an influence on perceptual priming in the neutral stories condition. This is in line with previous studies on the influence of cortisol on declarative memory processes showing that cortisol predominantly inhibits retrieval for emotionally arousing material [44], [45], [46], [47] (but see [19], [48] for contrary findings). It is also in line with findings from Luethi and colleagues [36] who showed that cortisol did not influence priming for neutral material.

Cortisol also did not influence recognition memory in the explicit memory test, even though cortisol is known to impact explicit memory processes. However, our participants only performed at chance level in the recognition memory test. Thus, our recognition memory test seems to have been too difficult to detect differences in memory performance between the conditions. Thus, we cannot interpret our null findings. Furthermore, due to the task difficulty of the recognition memory test, we cannot exclude that explicit memory for the neutral objects may have played a role in the perceptual priming task. There has been a debate in the literature on the influence of explicit memory processes on the performance of implicit memory tasks [49], such as the perceptual priming test employed in this study. However, previous studies using the perceptual priming paradigm [10], [11], [12] indicated that story context (neutral/”traumatic”) did not influence recognition performance. Nevertheless, our findings need to be replicated with a modified recognition task in order to analyze the effects of cortisol on explicit memory and the influence of explicit memory on performance in the perceptual priming task.

We used an analogue paradigm, which our participants perceived as moderately aversive. Even though ethical considerations limit the induction of trauma in the laboratory, there are analogue paradigms such as the trauma-film-paradigm, which are perceived as more aversive than our paradigm and which reliably lead to intrusive memories (for a review see [50]). However, our paradigm was designed to investigate perceptual priming in a “traumatic” context and the influence of cortisol on this memory mechanism and not to investigate intrusive memories per se. We could show that cortisol influences the retrieval of perceptually primed material in a “traumatic” context, a memory process that has been shown to underlie intrusive memories. In the next step it should be investigated whether cortisol is also able to inhibit intrusive memories as a response to an analogue trauma in an experimental setting.

Cortisol has been proposed as a pharmacological option to reduce intrusive reexperiencing symptoms in PTSD, because cortisol is known to inhibit memory retrieval. Another argument that has been brought up is that PTSD patients also show low endogenous cortisol levels. However, there is a recent debate on the existence of low cortisol levels in PTSD patients. While several recent studies still find low cortisol levels in PTSD patients (e.g. [26]), other studies indicate that low cortisol levels are a feature of trauma exposure rather than of PTSD (e.g. [27]). Still others show that the type of trauma (e.g. [29]) and comorbid depression (e.g. [24]) may play a role. Nevertheless, cortisol has also been shown to inhibit memory retrieval in a variety of studies with healthy controls (who did not have low basal cortisol levels) (for a review see [17]). Thus, cortisol might also prove to be useful in patients who do not show low endogenous cortisol levels.

It is important to note that PTSD is a severe psychological disorder, which is not only characterized by intrusive memories, but also by other symptoms such as feelings of shame, guilt, disgust, loss, and grief. We want to point out that we do not believe that cortisol may be a sole treatment option for PTSD. We rather believe that the memory modulating effects of cortisol might prove to be a useful adjunct to evidence based psychotherapy.

In summary, the current investigation demonstrates that cortisol does not only reduce the retrieval of neutral stimuli in explicit memory tests, but that it also reduces perceptual priming for stimuli occurring in a “traumatic” context. Thus, our study is the first to show that cortisol does inhibit memory processes linked to intrusions in PTSD, thereby supporting the idea that administration of cortisol might be an effective treatment strategy in reducing intrusive reexperiencing in PTSD.

## Supporting Information

Data S1 The original dataset is included as supporting information.
(XLSX)
